# Correspondence: Systematic review on the quality of randomized controlled trials in Saudi Arabia

**DOI:** 10.1016/j.conctc.2019.100483

**Published:** 2019-11-04

**Authors:** Khalid Aboalshamat

**Affiliations:** Head of Medicine and Medical Sciences Research Center, Deanship of Scientific Research, Umm Al-Qura University, Makkah, Saudi Arabia; Dental Public Health Division, Preventative Dentistry Department, College of Dentistry, Umm Al-Qura University, Makkah, Saudi Arabia

To the Editor,

I have read with great interest the systematic review that assessed the quality of randomized controlled trials (RCTs) in Saudi Arabia [1]. The article indicated that only 61 RCTs from Saudi Arabia were included in their systematic review after searching databases from inception until February 1, 2018. Furthermore, the authors concluded that no study was found to be with low risk, and the majority were classified as unclear risk according to the seven domains of the Cochrane Collaboration Risk of Bias Tool (CCRBT).

Despite the respected efforts of the authors of this review, some points are in need of verification before accepting the authors’ conclusions. These areas include the database search efforts, inter-rater reliability, and reporting of domain details.

First, the authors mentioned they used the following search terms: “Saudi Arabia,” “randomized controlled trial,” and “clinical trial” and indicated that 143 titles were identified in PubMed. In fact, the number of articles I found using the same keywords for the same period was much higher (1,538 titles), so perhaps the keywords might not have been used properly in the researching process. In fact, quickly skimming PubMed alone turned up several other important RCTs conducted in Saudi Arabia [[2], [3], [4], [5]]. None of these articles were among the 61 articles listed in the review. This jeopardizes the integrity of article selection in this systematic review, and because many important articles were not included, the authors are unable to make their conclusion.

Second, the authors highlighted that each article was assessed by two authors according to CCRBT domains, with the third author as referee in case of a dispute. However, there was no mention of the authors having formal training in using CCRBT guidelines to validate inter-rater reliability, which is an important factor for good assessments [6]. This is accentuated by the short period of time devoted to the review, according to the authors (March to April), which is linked to the next point.

Third, and most important, the authors gave descriptive statistics for the seven domains used to assess the 61 articles. However, there is a lack of information regarding each individual article's assessment. In other words, we cannot identify for each article which domain was classified as uncertain or high risk based on the information in the supplemental documents. This makes it difficult for another researcher to re-assess the 61 articles to replicate the findings. Such reports showing individual study assessments for each domain are found in similar systematic reviews that use the CCRBT [[7], [8], [9]].

Given these points, it is hard to accept the conclusion given in the referenced review. More rigorous study that includes all RCTs, with a high level of inter-rater reliability and appropriate reporting, is needed to validate the study's findings.

## Declaration of competing interest

None.
